# Regulatory Crosstalk by Protein Kinases on CFTR Trafficking and Activity

**DOI:** 10.3389/fchem.2016.00001

**Published:** 2016-01-20

**Authors:** Carlos M. Farinha, Agnieszka Swiatecka-Urban, David L. Brautigan, Peter Jordan

**Affiliations:** ^1^Faculty of Sciences, Biosystems and Integrative Sciences Institute, University of LisboaLisbon, Portugal; ^2^Department of Cell Biology, University of Pittsburgh School of MedicinePittsburgh, PA, USA; ^3^Children's Hospital of Pittsburgh of UPMC, University of Pittsburgh School of MedicinePittsburgh, PA, USA; ^4^Center for Cell Signaling and Department of Microbiology, Immunology, and Cancer Biology, University of Virginia School of MedicineCharlottesville, VA, USA; ^5^Department of Human Genetics, National Health Institute Dr Ricardo JorgeLisbon, Portugal

**Keywords:** CFTR, cystic fibrosis, phosphorylation, kinase, protein trafficking

## Abstract

Cystic Fibrosis Transmembrane Conductance Regulator (CFTR) is a member of the ATP binding cassette (ABC) transporter superfamily that functions as a cAMP-activated chloride ion channel in fluid-transporting epithelia. There is abundant evidence that CFTR activity (i.e., channel opening and closing) is regulated by protein kinases and phosphatases via phosphorylation and dephosphorylation. Here, we review recent evidence for the role of protein kinases in regulation of CFTR delivery to and retention in the plasma membrane. We review this information in a broader context of regulation of other transporters by protein kinases because the overall functional output of transporters involves the integrated control of both their number at the plasma membrane and their specific activity. While many details of the regulation of intracellular distribution of CFTR and other transporters remain to be elucidated, we hope that this review will motivate research providing new insights into how protein kinases control membrane transport to impact health and disease.

## CFTR role in health and disease

The Cystic Fibrosis Transmembrane Conductance Regulator (CFTR) protein is a polytopic integral membrane protein that functions as a cAMP- and phosphorylation-activated chloride (Cl^−^) channel at the apical surface of secretory epithelia. CFTR is a member of the ATP-Binding Cassette (ABC) transporter family, that hydrolyzes ATP to pump substrates, such as ions, vitamins, drugs, toxins, and peptides across biological membranes (Riordan, 2008). Mutations in the *CFTR* gene cause Cystic Fibrosis (CF), the most common autosomal recessive disorder among Caucasians. At present, more than 2000 mutations have been identified while 127 are confirmed as disease causing (Sosnay et al., 2013). Among these mutations, deletion of phenylalanine at position 508 in the polypeptide chain (F508del) is present in 85% of CF patients in at least one allele. F508del causes the majority of mutant CFTR protein to be retained in the endoplasmic reticulum (ER) with premature degradation by the ubiquitin-proteasome system (Riordan, 2008).

**Figure 1:**
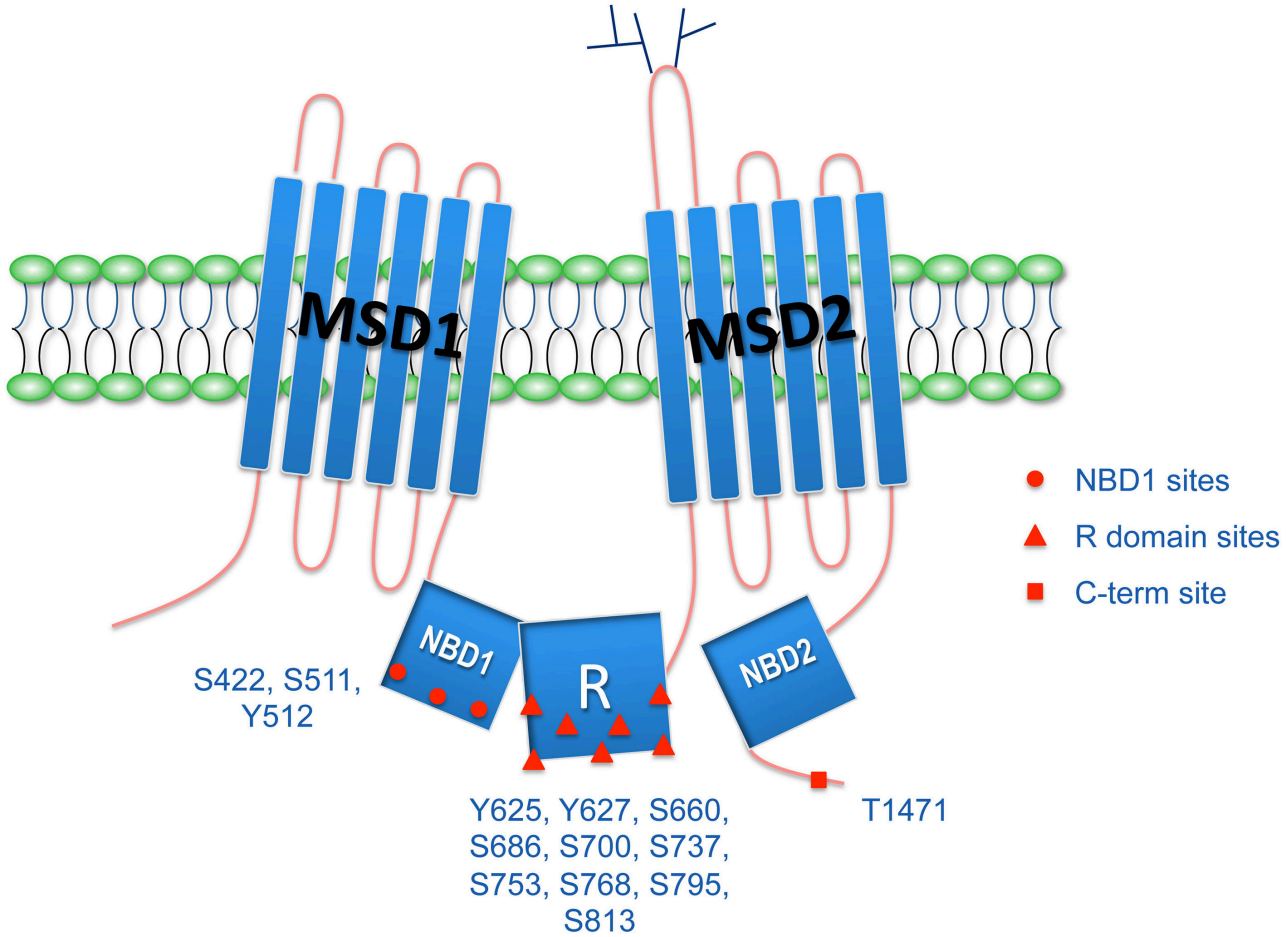
**CFTR structure and phosphorylation sites**. CFTR possesses two membrane spanning domains (MSD1 and MSD2), two nucleotide biding domains (NBD1 and NBD2), and the unique regulatory R domain.There are multiple phosphorylation sites in the R domain, but also in the NBD1 and in the C-terminus. Phosphorylation of CFTR regulates its biogenesis, interaction with other proteins, trafficking, and function.

Similarly to other ABC transporters, CFTR is composed of two nucleotide-binding domains (NBD), involved in channel regulation through ATP binding and hydrolysis, and two membrane-spanning domains (MSD), that form the pore of the channel. CFTR, however, is distinct in its structure as it possesses a regulatory (R) domain that contains multiple phosphorylation sites and a large proportion of charged amino acid residues (Figure 1; Higgins, 1992a). This distinct structural feature makes CFTR unique in the ABC transporter family and allows for tight regulation of its Cl^−^-secretory activity (Higgins, 1992b; Riordan, 2008).

The overall flux of Cl^−^ secretion through CFTR is the sum of the activity of each individual CFTR channel and the number of CFTR molecules present at the apical membrane. CFTR activity depends on the intrinsic structure of the CFTR protein, on several post-translational modifications, including phosphorylation, and on the binding and hydrolysis of ATP at the NBDs. The number of CFTR molecules at the PM results from a balance between anterograde trafficking (through which CFTR is delivered from the ER to the plasma membrane—PM), endocytosis (a process through which CFTR is retrieved from the membrane into vesicles), and recycling (with return of the internalized CFTR to the PM). Both, the activity of individual channels and the channel number at the PM are regulated by the interactions of CFTR with multiple protein partners and by post-translational modification (Sheppard et al., 1993; Zielenski, 2000; Wang and Linsdell, 2012).

## CFTR biogenesis and trafficking

Like most membrane proteins entering the secretory pathway, CFTR assembly begins with synthesis and folding in the ER, where it is core-glycosylated (Cheng et al., 1990). Co-translational folding of CFTR is an inefficient, slow, and complex process whereby the nascent polypeptide is concomitantly folded and inserted into the ER lipid bilayer (Farinha et al., 2002; Glozman et al., 2009). During the co- and post-translational folding, CFTR binds to several cytosolic and ER resident molecular chaperones as well as ubiquitin ligase enzymes (Meacham et al., 1999; Farinha et al., 2002). The glycans attached to CFTR are also responsible for the interaction between the protein and different lectins (in particular, calnexin), most of which participate in the ER quality control (ERQC). At this stage, misfolded CFTR is identified by the ERQC and degraded by the ubiquitin-proteasome pathway (UPP; Amaral, 2005; Farinha and Amaral, 2005). If correctly folded, CFTR proceeds to the secretory pathway through the Golgi complex where it undergoes further glycosylation and gradually attains its mature form. From the TGN, CFTR traffics to the PM where its pool is maintained by a balance between anterograde trafficking, endocytosis, recycling to the membrane and lysosomal degradation (Ameen et al., 2007).

F508del-CFTR can also accumulate at the PM when the trafficking defect is rescued by chemical, genetic, or physical strategies (e.g., small molecule compounds, second-site mutations, or low temperature, respectively; Amaral and Farinha, 2013). However, rescued F508del-CFTR exhibits defective channel gating—which can be partially due to defective phosphorylation (Pasyk et al., 2015)—and decreased stability. In fact, its biochemical half-life at the PM is about 4 h compared to that of wt-CFTR that exceeds 48 h (Heda et al., 2001). This instability is due to more rapid endocytosis of mutant CFTR protein and/or its selective targeting for lysosomal degradation (Swiatecka-Urban et al., 2005). Moreover, F508del-CFTR recycling is attenuated by nearly five-fold as compared with wt-CFTR, suggesting that misfolding of CFTR has also a major impact on the sequestration of CFTR at the early endosome (Sharma et al., 2004).

## CFTR phosphorylation is needed for its channel activity

CFTR channel function depends on the conformational changes driven by ATP binding and hydrolysis at the heterodimer NBD1:NBD2, shifting the molecule between the open and closed states (Anderson et al., 1991). The transition in conformation is however initiated by protein kinase A (PKA)-dependent phosphorylation of the R domain. The open probability of the channel is dependent on R domain phosphorylation at multiple sites (Figure 1), reflecting a balance between protein kinase and phosphatase activity (Cheng et al., 1991). Although CFTR is primarily phosphorylated by PKA, other kinases have been described to activate (cGMP-dependent protein kinase, Src, and proline-rich tyrosine kinase Pyk2; Billet et al., 2015a,b), to inhibit [AMP-dependent protein kinase (AMPK); Hallows et al., 2000], or to have a dual effect on the channel activity [PKA and protein kinase C (PKC); Jia et al., 1997; Chappe et al., 2004] depending on the consensus site phosphorylated (Berger et al., 1993; Alzamora et al., 2011). Among several phosphorylation sites in the R domain, two in particular—S737 and S768—have a phospho-dependent inhibitory effect on the CFTR channel function (Wilkinson et al., 1997). Phosphorylation of these inhibitory sites by AMPK inhibits CFTR mediated Cl^−^ secretion by maintaining the channel in a closed state (Kongsuphol et al., 2009). Recent *in vitro* work demonstrated that the S737 site is also a preferred substrate for Lemur Tyrosine kinase (LMTK2; Wang and Brautigan, 2006), as discussed below, but the tissue specificity and hierarchy of the regulatory mechanisms involving either AMPK or LMTK2 remain to be determined. In the following, we will focus on recent evidence demonstrating how kinases regulate CFTR biogenesis and trafficking, besides the well-characterized role in activating CFTR, briefly described above.

## CK2 as a regulator of CFTR biogenesis and function

Casein Kinase II (CK2) is a ubiquitous, pleiotropic, and constitutively active Ser/Thr protein kinase with a heterotetrameric structure, where the holoenzyme contains two α (catalytic) subunits offering the potential to simultaneously phosphorylate different substrates. Additionally, CK2 has two β regulatory, polyamine-binding subunits (Meggio and Pinna, 2003). CK2 is considered a master regulator that controls protein expression, cell signaling, and ion channel activity by coordinating the function of other kinases (Meggio and Pinna, 2003). CK2 has been reported to regulate protein trafficking at multiple stages in the secretory pathway. Phosphorylation by CK2 regulates the interaction of PM proteins with adaptors, such as arrestins, thus controlling internalization of transporters such as the Na+/H+ exchanger NHE5 and receptors, including thyrotropin- and gonadotropin-releasing hormone receptors (Hanyaloglu et al., 2001; Lukashova et al., 2011). The kinase has also been reported to phosphorylate proteins involved in cell-to-cell junctions, such as E-cadherin whose phosphorylation by CK2 leads to its stabilization at the PM and to the strengthening of adherens junctions in keratinocytes (Serres et al., 2000). In addition, CK2 controls protein trafficking adaptors, as it has been reported to phosphorylate Sec31, thus regulating the duration of COPII vesicle formation, and p115, a vesicle-tethering factor essential for ER-to-Golgi transport (Dirac-Svejstrup et al., 2000; Koreishi et al., 2013). CK2 also regulates lysosomal hydrolase trafficking and sorting of proteins in the transGolgi network/endosomal system (Stockli et al., 2004; Scott et al., 2006).

CK2 has been shown to regulate both CFTR biogenesis and function (Treharne et al., 2009; Luz et al., 2011). The co-localization of CK2 with wt-CFTR raised the possibility that the kinase regulates CFTR channel function in apical membranes of airway epithelial cells (Treharne et al., 2009). This was confirmed by the finding that an inhibitor of CK2 attenuated CFTR-dependent Cl^−^ transport in different model systems, including mouse colon (Treharne et al., 2009; Luz et al., 2011). This evidence was complemented with the *in vitro* CK2 phosphorylation of CFTR at S422 (Pagano et al., 2008). Although several consensus sites for CK2 exist in CFTR, the region near to F508 (namely the putative site S511) is not phosphorylated by CK2. Instead, this CFTR region acts to allosterically control both the isolated catalytic subunit and the holoenzyme of CK2 (Pagano et al., 2010). This was the first evidence demonstrating a mutual CK2-CFTR interplay (Pagano et al., 2010). Specifically, CK2 regulates the CFTR channel function, CK2 is modulated by CFTR—being functionally perturbed by the F508del CFTR mutation. Subsequently the close interplay between CFTR and CK2 was confirmed by another study describing that CK2 inhibition reduced the difference between the phosphoproteome resulting from the F508del CFTR gene mutation (Venerando et al., 2011).

The role of CK2 in CFTR regulation was reinforced by the observation that its pharmacological inhibition decreased processing of wt-CFTR (Luz et al., 2011). This effect may involve reduced phosphorylation of CFTR by CK2, previously shown to occur at S422 (Pagano et al., 2008), or may occur indirectly through other targets of CK2 that act as CFTR-associated proteins. Another mechanism of regulation may occur through phosphorylation of T1471 at CFTR C-terminus, a region involved in the control of multiple protein-protein interactions that are critical for CFTR stability at the PM (Luz et al., 2011). This T1471 site is homologous to T1467 in murine CFTR that is readily phosphorylated by CK2 *in vitro* (Venerando et al., 2013). Furthermore, a 42 amino acid peptide encompassing the C-terminal segment of human CFTR is readily phosphorylated at T1471 with favorable kinetics by the CK2 holoenzyme, but neither by its isolated catalytic subunit nor by other acidophilic Ser/Thr kinases such as CK1, PLK2/3, GCK/FAM20C (Venerando et al., 2013). These putative CK2 phosphorylation sites also impact the degradation pattern of CFTR—in fact, cells generate endogenous CFTR fragments whose combined fingerprint differs not only after F508 deletion, but also after mutation of residues S511 and T1471 that are relevant to the regulatory interactions of CFTR with protein kinase CK2 (Tosoni et al., 2013).

Recently, it was shown that CK2 inhibition is involved in the mechanism through which epigallocatechin gallate (EGCG), a green tea flavonoid, prolongs the rescue of F508del-CFTR PM localization by cysteamine (De Stefano et al., 2014). This effect was observed not only in cell lines but also in a mouse model and in nasal cells from F508del-CFTR homozygous patients and demonstrated for the first time that the kinase inhibition may impact on the rescue of mutant CFTR (De Stefano et al., 2014).

In summary (Table 1), strong evidence exists that CK2 phosphorylates different sites in CFTR to control its function and biogenesis. The CK2-CFTR interaction allows an allosteric control of the kinase and regulates CFTR degradation, i.e., fragmentation pattern. The differential effect of wt- and F508del-CFTR interplay with CK2 may provide an additional target for modulation of CFTR trafficking and function in CF (Venerando et al., 2011; De Stefano et al., 2014). The recent data that CK2 is a key player in the correction of the basic defect in patients bearing the F508del mutation further strengthens this premise (De Stefano et al., 2014).

**Table 1:** **The CFTR-CK2 crosstalk**.

CK2 modulates CFTR	Promotes CFTR processing (Luz et al., 2011)
	Promotes CFTR Cl^−^ channel function (Treharne et al., 2009)
	Mediates F508del-CFTR rescue by cysteamine (De Stefano et al., 2014)
	Modulates CFTR degradation (Tosoni et al., 2013)
CFTR modulates CK2	Allosterically regulates CK2 activity (Pagano et al., 2008, 2010; Venerando et al., 2011, 2013)

## SYK controls CFTR membrane levels

A second kinase that regulates CFTR trafficking is Spleen Tyrosine Kinase (SYK). SYK is a cytoplasmic non-receptor tyrosine kinase best known for its pro-inflammatory role in immunoreceptor signaling in leukocytes (Mócsai et al., 2010). SYK has been reported to phosphorylate CFTR at residue Y512 leading to a decrease in PM levels of CFTR (Luz et al., 2011; Mendes et al., 2011).

The first evidence linking CFTR and SYK suggested possible functional interactions between SYK and CK2 in regulating CFTR function. In particular, CK2 was found to phosphorylate CFTR peptides corresponding to the sequence PGTIKENIIFGVSY_512_DEYRYR only when residue Y512 was substituted with phosphotyrosine, suggesting a hierarchical interplay between the two kinases that requires priming phosphorylation of CFTR Y512 by SYK, described both *in vitro* and *in vivo* (Pagano et al., 2008, 2010; Luz et al., 2011; Mendes et al., 2011).

Inhibition of SYK (or mutation of the potential SYK-phosphorylation site in CFTR) was shown to strongly augment Cl^−^ currents in *Xenopus* oocytes, even those produced by F508del-CFTR (Luz et al., 2011). The phosphorylation of Y512 is probably responsible for the interaction of SYK and CFTR in human airway epithelial cells and leads to reduction in CFTR at the PM. The effect was further demonstrated in CFTR variants that mimic either a phosphorylated (Y512E) or non-phosphorylated (Y512F) condition, with the former leading to a reduction and the latter to an increase in the steady-state levels of CFTR in the PM. However, the mechanistic details by which SYK phosphorylation regulates CFTR levels in the PM have not been elucidated. It is possible that SYK phosphorylation of CFTR enhances its interaction with the kinase, as SYK contains two SH2 domains (Mócsai et al., 2010). SH2 domains dock to phosphorylated tyrosine residues on proteins (Mócsai et al., 2010), and if SYK affects CFTR by such mechanism, the kinase may function as an adaptor to regulate CFTR trafficking. It remains unclear whether phosphorylation of Y512 can occur through other kinases, as it was shown that the Src family kinase Lyn phosphorylates this residue in a peptide encompassing CFTR residues 500–523 *in vitro* (Cesaro et al., 2013).

Such a role of SYK in regulating protein trafficking has been reported previously for other substrates. SYK can bind through its SH2 domain to the phosphorylated form of the membrane protein TGN38 and this association leads to increased PM expression of TGN38, reinforcing that tyrosine-based motifs may play a broader role in integrating trafficking and signaling events (Stephens and Banting, 1997). Other data support a role for SYK in the regulation of protein trafficking. For example, SYK is essential for the trafficking of engaged high affinity IgE receptor (FcεRI) to a degradative compartment in mast cells. In this process, SYK phosphorylates the hepatocyte growth factor-regulated tyrosine kinase substrate (Hrs), an effector that functions as critical regulator for FcεRI entry into lysosomes, providing an example on how SYK orchestrates the crosstalk between endocytosis and signaling (Gasparrini et al., 2012). Moreover, phosphorylated SYK recruits and activates multiple downstream signaling molecules, including the small GTPases Rac1 and Cdc42 (Greenberg, 1999), the former of which was recently shown to play a role in CFTR trafficking and membrane anchoring (Moniz et al., 2013).

**Figure 2:**
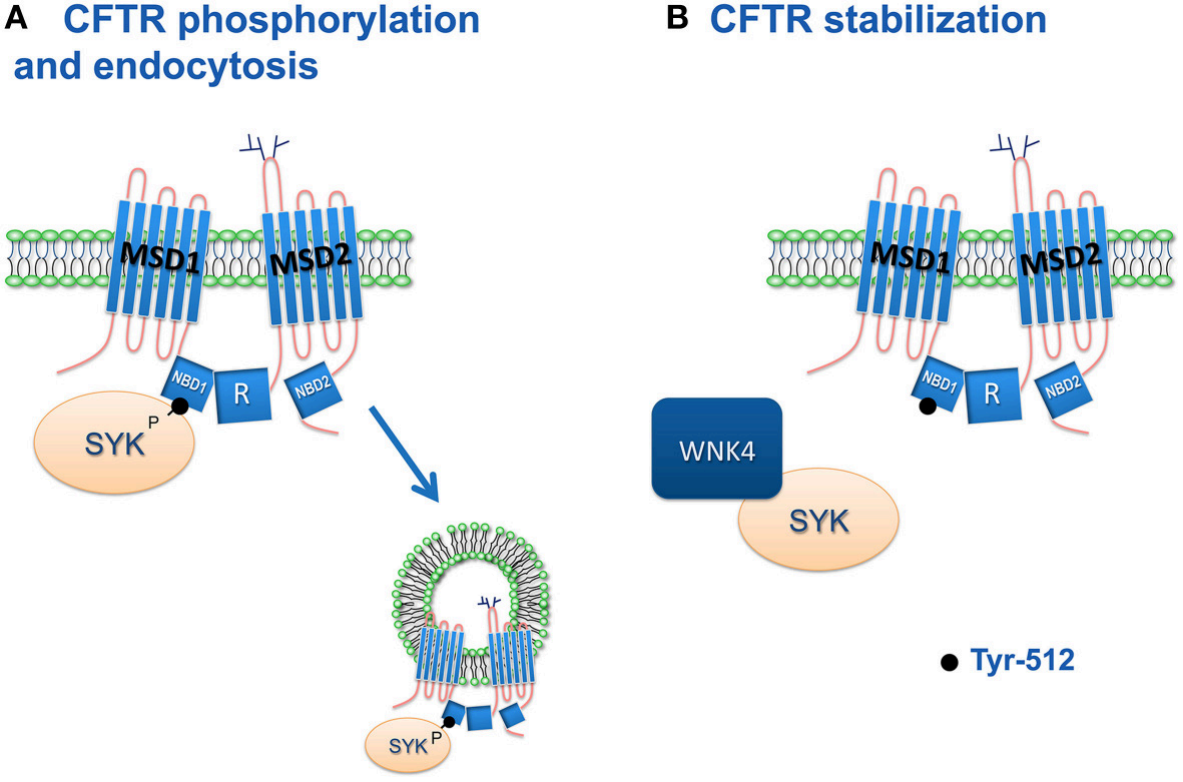
**Proposed model for the role of SYK in regulating CFTR plasma membrane levels**. **(A)** Activated SYK phosphorylates CFTR Y512 and triggers its endocytosis. **(B)** WNK4 sequesters and inhibits SYK, thus stabilizing CFTR channels at the PM and increasing CFTR mediated Cl^−^ transport (Mendes et al., 2011).

Current data show that phosphorylation of CFTR at Y512 by SYK decreases the PM CFTR abundance. Thus, SYK inhibition may stabilize the PM wt-CFTR as well as the rescued F508del-CFTR (Figure 2). SYK knockdown in airway epithelial cells downregulates IL-6 and ICAM-1 (Ulanova et al., 2005). These proinflammatory mediators are elevated in CF patients (Nixon et al., 1998). Thus, SYK emerges as a potential target to stabilize rescued mutant CFTR and attenuate the proinflammatory mediators in CF.

## WNKs crosstalk with SYK to regulate CFTR trafficking

The human subfamily of WNK (with-no-lysine) protein kinases comprises four members, WNK1 through 4 (Veríssimo and Jordan, 2001; Manning et al., 2002; Hanks, 2003), which regulate the function and trafficking of different transporters. Mutations in the *WNK1* and *WNK4* genes were initially discovered to cause Gordon's syndrome (or pseudo-hypoaldosteronism type II, PHA-II), a rare familial form of hypertension (Wilson et al., 2001) caused by renal salt retention. Subsequently, evidence has been provided that WNK1 and WNK4 regulate the transport activity of the renal co-transporter NCC by activating other protein kinases, SPAK (STE20/SPS1-related, proline alanine-rich kinase) or OSR-1 (Oxidative-stress-responsive kinase 1), which in turn directly phosphorylate the N-terminal serine residues in the co-transporters (Gamba, 2005; Kahle et al., 2008).

WNK kinases are co-expressed with CFTR in a variety of tissues, including ciliated epithelial cells in the lung (Choate et al., 2003; Yang et al., 2007). WNK1 and WNK4 inhibit CFTR chloride channel activity in *Xenopus* oocytes, but the mechanisms appear to be different as the two kinases exhibit additive CFTR inhibition. The inhibitory effects of WNK1 are kinase-dependent and do not affect CFTR abundance at the PM, whereas the inhibitory effects of WNK4 are kinase-independent and associated with a reduction in CFTR at the PM. In *Xenopus* oocytes mutant WNK4 exhibits enhanced inhibition of CFTR activity when compared to wt-WNK4 (Yang et al., 2007). This is in agreement with the observation that PHA-II patients with WNK4 mutations exhibit changes in epithelial potential difference and conductance that resemble mild CF, which could be explained by the ability of mutant WNK4 to suppress the the epithelial sodium channel (ENaC) or CFTR in nasal epithelia and sweat ducts of PHA-II patients (Farfel et al., 2005; Yang et al., 2007).

The effects of WNK4 on CFTR activity are reminiscent of the effects of WNK1, WNK3, and WNK4 on the activity of the Cl^−^/${\text{HCO}}_{3}^{-}$ exchanger SLC26A9 and of WNK1 and WNK4 effects on the renal outer medullar potassium 1 (ROMK1) channel (Kahle et al., 2003; Lazrak et al., 2006; He et al., 2007). The effects of different WNK kinases on these three transporters are kinase-independent and associated with a change in protein abundance at the PM. Therefore, WNKs could act here as scaffolds that recruit other proteins that regulate the activity and/or PM expression of these channels. Such an effect was reported in a recent study in renal distal convoluted tubule cells, where angiotensin II and WNKs regulate the sodium-chloride co-transporter NCC in a dual mode: a rapid response within 15 min is mediated by WNK4-dependent trafficking of NCC to the PM, but sustained stimulation for over 60 min activates WNK4/SPAK-dependent NCC phosphorylation and transporter activation (Ko et al., 2015). Similarly, WNK1 and WNK4 have been shown to stimulate clathrin-dependent endocytosis of ROMK1, thus inhibiting potassium secretion (Kahle et al., 2003; Cope et al., 2006; Lazrak et al., 2006; He et al., 2007). A variety of other reports described that WNKs also regulate PM abundance of a variety of extra-renal ion transporters, including the potassium-chloride co-transporter KCC, the transient receptor potential cation (calcium) channel subfamily V members 4 and 5 (TRPV4 and TRPV5; Kahle et al., 2004, 2006, 2008; Gamba, 2005), and ENaC.

Some mechanistic insights into how WNKs regulate PM expression have been obtained. WNK4 inhibited the renal co-transporter NCC (Cai et al., 2006; Subramanya et al., 2009) but also the potassium channel Maxi K (Subramanya et al., 2009; Zhuang et al., 2011) by redirecting the channels to lysosomal degradation. For example, WNK4 associates with wt- as well as Liddle's mutated ENaC and enhances ENaC internalization independent of Nedd4-2-mediated ENaC ubiquitination by an unknown mechanism (Yu et al., 2013). WNK4 also inhibits protein delivery and attenuates PM-targeting of NCC through binding to syntaxin13 and inhibiting the SNARE complex formation with VAMP2 in recycling and sorting endosomes (Chung et al., 2013). In the case of TRPV5, WNK4 decreased PM abundance of TRPV5 by enhancing its caveolin-mediated endocytosis (Cha and Huang, 2010). Additional support demonstrating the role of WNKs in endocytosis comes from a genome-wide RNAi screen. In this screen, virus entry was used to measure clathrin-mediated (vesicular stomatitis virus—VSV) or caveolin-dependent (simian virus—SV40) endocytosis. It was found that WNK4 interfered with the clathrin-mediated VSV entry whereas WNK2 inhibited caveolin-mediated SV40 uptake (Pelkmans et al., 2005). However, no further mechanistic details were determined.

WNK4 was also shown to modulate the levels of CFTR at the PM. In CFTR trafficking, WNK4 acts antagonistically to SYK and promotes PM expression of CFTR (Figure 2). WNK kinases are activated by changes in extracellular osmolarity (Kahle et al., 2008). This regulatory mechanism may be used by WNK4 to adjust CFTR function by sensing the osmolarity of the airway surface liquid. This inhibition of SYK promoted by WNK4 seems to be more dependent on its interaction with SYK rather than a phosphorylation, as the inhibition is also observed with the kinase dead WNK4 (Mendes et al., 2011). This is in agreement with the inhibitory effects of WNK4 on PM ROMK1 (Kahle et al., 2003) and EnaC (Ring et al., 2007). The antagonistic effects of WNK4 and SYK on CFTR may also involve endocytosis although the mechanism has not been elucidated. If SYK phosphorylates CFTR and triggers its endocytosis, WNK4 could sequester SYK and increase retention of CFTR at the PM.

## LMTK2 as a regulator of CFTR membrane localization and function

The fourth protein kinase with a defined role in regulation of CFTR trafficking and PM stability is Lemur Tyrosine Kinase 2 (LMTK2). LMTK2 is a member of the lemur family of membrane-anchored kinases with multiple aliases such as kinase/phosphatase/inhibitor-2 (KPI2), brain-enriched kinase (BREK), apoptosis-associated tyrosine kinase (AATYK2), or cyclin-dependent kinase-5 (cdk5/p35)-regulated kinase, describing different functions and tissue localizations of the protein. Despite the original prediction of a dual-specificity serine-threonine/tyrosine kinase, biochemical studies have shown that purified LMTK2 kinase domain phosphorylates only serine and threonine, but not tyrosine residues (Wang and Brautigan, 2002, 2006; Kawa et al., 2004). LMTK2 forms a regulatory network with several cytosolic proteins via its long C-terminal tail domain that prompted naming the protein after the lemur, a long-tailed Madagascar primate (reviewed in Rattray, 2012). The N-terminal transmembrane domain anchors LMTK2 at the PM and is followed by the kinase domain (Wang and Brautigan, 2002; Marchler-Bauer et al., 2003, 2013; Nixon et al., 2013). The role of LMTK2 in intracellular trafficking was first described in neuronal and muscle tissues (Lew et al., 1994; Tsai et al., 1994; Fu et al., 2001; Chibalina et al., 2007; Inoue et al., 2008; Manser et al., 2012). Subsequently, LMTK2 was shown to facilitate endocytosis and decrease PM abundance of CFTR in human bronchial epithelial (HBE) cells (Luz et al., 2014).

The site for CFTR phosphorylation by LMTK2 is the inhibitory PKA/AMPK-site S737 (Wang and Brautigan, 2006). In HBE cells, including primary differentiated HBE cells, endogenous LMTK2 phosphorylates CFTR-S737, and decreases CFTR mediated Cl^−^ secretion by inducing endocytosis and inhibiting PM accumulation of CFTR (Luz et al., 2014). The authors presented several lines of evidence supporting these conclusions. First, endogenous LMTK2 accumulated at the apical PM of polarized HBE cells and co-immunoprecipitated with CFTR, indicating that LMTK2 and CFTR physically associate within this membrane domain. Next, inactivation of LMTK2 by mutagenesis of the kinase domain reduced phosphorylation of CFTR-S737 in HBE cells. Compared to controls, LMTK2 knockdown by siRNA or substitution of the LMTK2 phosphorylation site CFTR-S737 by a non-phosphorylated residue increased the steady-state PM abundance of CFTR and decreased its endocytosis. Conversely, inactivation of the LMTK2 kinase by mutagenesis increased the steady-state PM abundance of CFTR and decreased CFTR endocytosis compared to control. LMTK2 also interacts with myosin VI and together these proteins mediate endocytic trafficking (Chibalina et al., 2007; Inoue et al., 2008; reviewed in Rattray, 2012). It had been shown that myosin VI facilitates CFTR endocytosis (Swiatecka-Urban et al., 2004). Using truncated LMTK2 proteins without the myosin VI binding domain or the tail domain allowed the authors to demonstrate that LMTK2 kinase specifically regulates the PM accumulation and endocytosis of CFTR (Luz et al., 2014).

**Figure 3:**
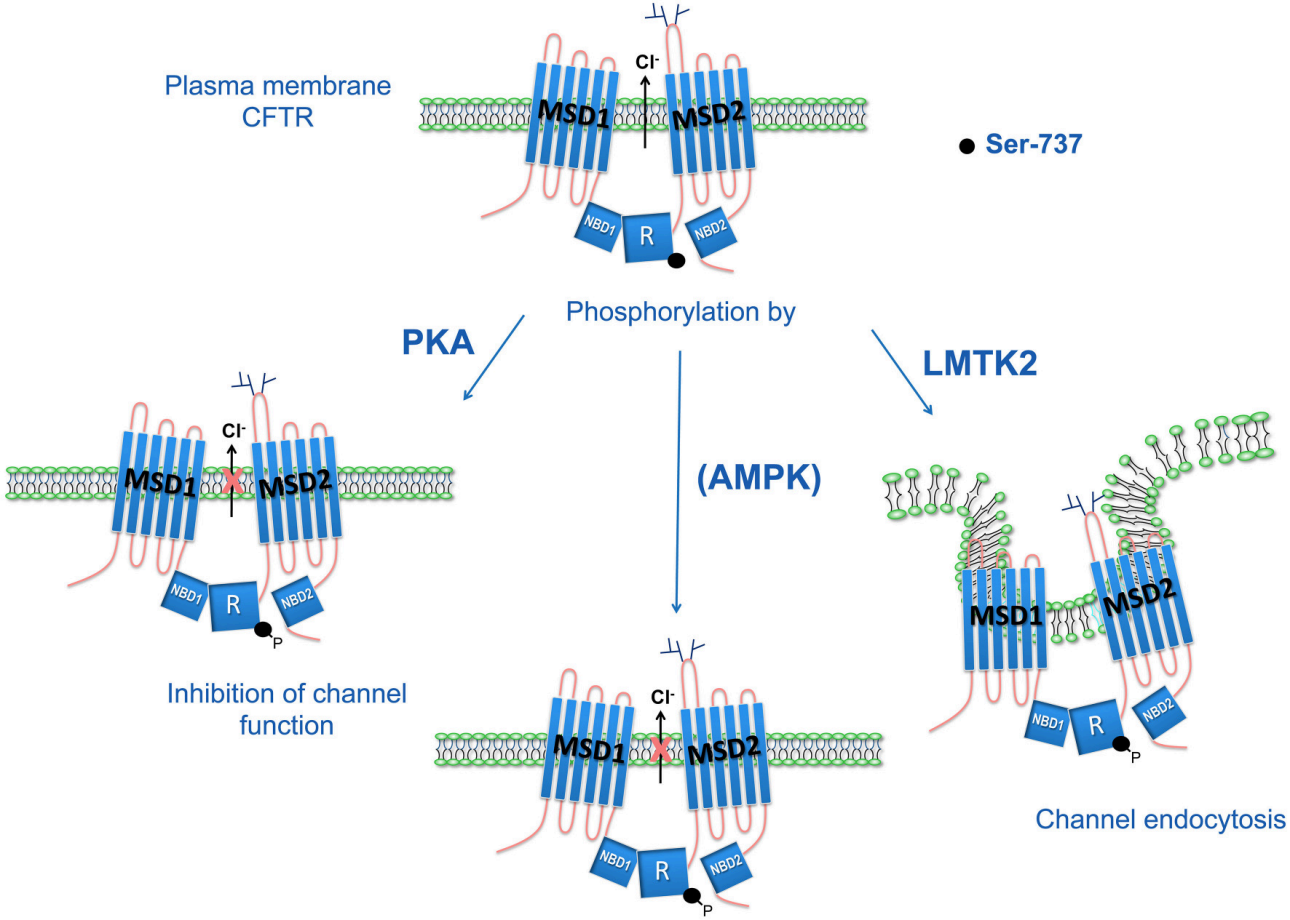
**Phospho-dependent inhibitory effects of CFTR-S737 on CFTR mediated Cl^−^ secretion**. Serine-737 (S737) in CFTR regulatory (R) domain has a phospho-dependent inhibitory effect on CFTR-mediated Cl^−^ secretion. The PKA-mediated phosphorylation of S737 inhibits CFTR Cl^−^ channel function. AMPK also phosphorylates the S737 site and inhibits CFTR mediated Cl^−^ secretion by maintaining CFTR channels in a closed state. This mechanism was proposed to play a role in non-stimulated epithelia with high baseline AMPK activity (Kongsuphol et al., 2009). LMTK2 phosphorylation of S737 facilitates CFTR endocytosis, reduces the PM density of CFTR Cl^−^ channels, and ultimately reduces CFTR mediated Cl^−^ secretion (Luz et al., 2014).

It remains unknown whether LMTK2 phosphorylation of CFTR-S737 may also inhibit CFTR channel function in addition to reducing the PM density of CFTR. Furthermore, the effects of AMPK- and PKA-mediated phosphorylation of CFTR-S737 on the PM CFTR abundance are unknown. Hence, it remains to be determined whether the phospho-dependent inhibitory effects on CFTR-S737 are differentially mediated by each of the three kinases, PKA, AMPK, and LMTK2 (Figure 3).

LMTK2 likely plays a physiologically relevant role in regulating the inhibitory CFTR-S737 site in HBE cells because, similar to mutagenesis of the S737 site, LMTK2 knockdown produced a two-fold increase in the PM abundance of wt-CFTR and a three-fold decrease in CFTR endocytosis. When compared to the LMTK2 knockdown, the kinase-dead LMTK2 fragment produced lesser effect on the PM abundance and endocytosis of CFTR, possibly because endogenous LMTK2 diminished the effects of the kinase deficient fragment when compared to LMTK2 knockdown (Luz et al., 2014). It has been proposed that AMPK activity is increased in non-stimulated epithelia, leading to phosphorylation of S737, and inhibition of CFTR channel function (Kongsuphol et al., 2009). Although it is a plausible explanation, little data exist to support this prediction. Similarly, increased activity of LMTK2 at the apical PM was proposed to inhibit CFTR-mediated Cl^−^ secretion at least in part by attenuating the PM abundance of CFTR channels; however, the mechanism through which LMTK2 activity is regulated at the apical PM in HBE cells is unknown (Luz et al., 2014). LMTK2 function can be affected by LMTK2 gene polymorphisms or by protein kinase cdk5/p35, neither of which are known to affect CFTR (Harries et al., 2010; Seo et al., 2012). Mutations in the LMTK2 gene have also been associated with lung adenocarcinoma in smokers (Seo et al., 2012), an interesting observation since cigarette smoke is a negative regulator of CFTR (reviewed in Rab et al., 2013). The mechanisms of CFTR inhibition by cigarette smoke are incompletely defined so that examining the effects of smoke exposure on LMTK2 activity may increase our understanding of the environmental toxin's effects on CFTR function. Thus, far, it is unknown whether the CF lung environment or CF mutations affect LMTK2 expression and function.

LMTK2 knockdown increased CFTR mediated Cl^−^ secretion in cells expressing wt-CFTR (Luz et al., 2014). This effect was also observed in cells expressing F508del-CFTR and correlated with an increase in the amount of corrector VX-809-rescued F508del-CFTR (mature band C). The effect of VX-809 was small but similar to that observed in previous studies (Van Goor et al., 2011; Cihil et al., 2013; Holleran et al., 2013). LMTK2 knockdown differentially affected the biochemical and functional rescue of F508del-CFTR where a 10% increase in CFTR band C abundance translated into a 50% rise of the forskolin/IBMX-stimulated short-circuit current. Clinical studies have already shown that targeted monotherapy with the CFTR correctors in patients homozygous for F508del mutation is promising but still inadequate, likely because of decreased function or reduced PM stability of the pharmacologically-rescued F508del-CFTR (Van Goor et al., 2011, 2014; Clancy et al., 2012; Molinski et al., 2012). Hence, attenuating LMTK2 phosphorylation of F508del-CFTR may play a role together with CFTR correctors and potentiators to maximally rescue F508del-CFTR function in CF patients. LMTK2 knockdown in immortalized HBE cells did not increase the steady-state abundance of partially glycosylated F508del-CFTR band B and it did not rescue F508del-CFTR in the absence of VX-809. These data suggest that the effects of LMTK2 knockdown on CFTR in these cells were mediated via alterations of post-maturational trafficking of CFTR during endocytic uptake and not during the biosynthetic processing of CFTR. It remains to be determined whether LMTK2 knockdown has a similar effect in primary differentiated HBE cells.

In summary, published data support a model whereby phosphorylation of S737 by PKA or AMPK closes CFTR channels, while phosphorylation of S737 by LMTK2 reduces the PM density of CFTR. More research is needed to determine the specific contribution of the kinases to regulation of CFTR function in different tissues and under different physiological and disease states. Because rescued F508del-CFTR has decreased PM stability, targeting LMTK2 phosphorylation of CFTR may offer a novel approach to investigate the stability defect and to design pharmacological approaches to stabilize rescued F508del-CFTR. These findings may also point to a new direction in elucidating the mechanisms of decreased CFTR function in lung diseases resulting from cigarette smoke inhalation.

## Conclusion

CFTR processing, trafficking, and activation are regulated by multiple protein-protein interactions. While interactions resulting in phosphorylation at the CFTR R domain regulate channel opening, other interactions at the CFTR termini modulate CFTR stability at the PM. For example, syntaxins interact with the CFTR N-terminal tail and PDZ-proteins bind to the PDZ-interacting domain at the CFTR C-terminal tail. The multiprotein scaffolds anchor CFTR to the cytoskeleton and allow CFTR to regulate other channels. Recent evidence, reviewed here, has identified novel protein partners, including the kinases CK2, SYK, WNK4, and LMTK2 regulating the biogenesis, turnover, and trafficking of CFTR. Such evidence increases our understanding of the complex CFTR interactome and points to novel directions in search of additional therapeutic targets to rescue F508del-CFTR and to increase the efficiency of current generation CFTR modulators.

## Author contributions

CF, AS, DB, and PJ discussed and wrote the manuscript.

### Conflict of interest statement

The authors declare that the research was conducted in the absence of any commercial or financial relationships that could be construed as a potential conflict of interest.

## Abbreviations

AATYK2: apoptosis-associated tyrosine kinase
AMPK: AMP-dependent kinase
BREK: brain-enriched kinase
cdk5: cyclin-dependent kinase-5
CF: Cystic fibrosis
CFTR: Cystic fibrosis transmembrane conductance regulator
CK2: casein kinase 2
ENaC: epithelial sodium channel
HBE: human bronchial epithelial
LMTK2: Lemur tyrosine kinase
MSD: membrane spanning domain
NBD: nucleotide binding domain
NCC: sodium-chloride co-transporter
OSR-1: oxidative-stress-responsive kinase 1
PHA-II: pseudo-hypoaldosteronism type II
PKA: protein kinase A
PKC: protein kinase C
Pyk: proline-rich tyrosine kinase
R: regulatory domain
KPI-2: kinase/phosphatase/inhibitor-2
ROMK1: renal outer medullary potassium channel
SLC26A9: solute carrier 26A9
SPAK: STE20/SPS1-related, proline alanine-rich kinase
SYK: spleen tyrosine kinase
TRPV: transient receptor potential cation channel subfamily V
WNK: with-no-lysine kinase.
